# Improvement of non-alcoholic steatohepatitis by hepatocyte-like cells generated from iPSCs with Oct4/Sox2/Klf4/Parp1

**DOI:** 10.18632/oncotarget.23603

**Published:** 2018-01-02

**Authors:** Yueh Chien, Chi-Shuan Huang, Hsin-Chi Lin, Kai-Hsi Lu, Ping-Hsing Tsai, Ying-Hsiu Lai, Kuan-Hsuan Chen, Shou-Dong Lee, Yi-Hsiang Huang, Chien-Ying Wang

**Affiliations:** ^1^ Institute of Pharmacology, National Yang-Ming University, Taipei, Taiwan, ROC; ^2^ Institute of Clinical Medicine, National Yang-Ming University, Taipei, Taiwan, ROC; ^3^ Faculty of Medicine, National Yang-Ming University, Taipei, Taiwan, ROC; ^4^ School of Medicine, National Yang-Ming University, Taipei, Taiwan, ROC; ^5^ Division of Colorectal Surgery, Department of Surgery, Cheng-Hsin General Hospital, Taipei, Taiwan, ROC; ^6^ Division of Gastroenterology, Department of Internal Medicine, Cheng-Hsin General Hospital, Taipei, Taiwan, ROC; ^7^ Department of Medical Research and Education, Cheng-Hsin General Hospital, Taipei, Taiwan, ROC; ^8^ Division of Gastroenterology, Department of Internal Medicine, Taipei Veterans General Hospital, Taipei, Taiwan, ROC; ^9^ Department of Medical Research and Education, Taipei Veterans General Hospital, Taipei, Taiwan, ROC; ^10^ Department of Pharmacy, Taipei Veterans General Hospital, Taipei, Taiwan, ROC; ^11^ Division of Trauma, Department of Emergency Medicine, Taipei Veterans General Hospital, Taipei, Taiwan, ROC

**Keywords:** non-alcoholic steatohepatitis, induced pluripotent stem cells

## Abstract

The prevalence of nonalcoholic fatty liver disease (NAFLD) is usually increased with age. Non-alcoholic steatohepatitis (NASH), a serious form of NAFLD, may lead to cirrhosis and end-stage liver diseases. Induced pluripotent stem cells (iPSCs) hold promising potential in personalized medicine. Although obviation of c-Myc reduces tumorigenic risk, it also largely reduced the generation of iPSCs. Recently, Poly(ADP-ribose) polymerase 1 (Parp1) has been reported to enhance cell reprogramming. In this study, we demonstrated that forced expression of Oct4/Sox2/Klf4/Parp1 (OSKP) effectively promoted iPSC generation from senescent somatic cells from 18-month-old mouse. The iPSCs presented regular pluripotent properties, ability to form smaller teratoma with smaller size, and the potential for tridermal differentiation including hepatocyte-like cells (OSKP-iPSC-Heps). Resembled to fetal hepatocytes but not senescent hepatocytes, these OSKP-iPSC-Heps possessed antioxidant ability and were resistant to oxidative insult induced by H_2_O_2_ or exogenous fatty acid. Intrasplenic transplantation of OSKP-iPSC-Heps ameliorated the triglyceride over-accumulation and hepatitis, prevented the production of inflammatory cytokines and oxidative substances, and reduced apoptotic cells in methionine/choline-deficient diet (MCDD)-fed mice. In conclusion, we demonstrated that Parp-1 promoted iPSC generation from senescent cells, which can be used for the treatment of NASH after hepatic-specific differentiation. These findings indicated that patient-derived iPSC-Heps may offer an alternative option for treatment of NASH and NASH-associated end-stage liver diseases.

## INTRODUCTION

Non-alcoholic fatty liver disease (NAFLD), referring to the fat deposition in the liver independent of excessive alcohol use, has become a very common liver disease worldwide. The prevalence of NAFLD in the population of Western countries is 20–30% [1] and usually increased with age. About 2–3% of the general population is estimated to have non-alcoholic steatohepatitis (NASH), the most extreme form of NAFLD. Approximately 15–25% NASH patients will develop liver fibrosis, and once cirrhosis occurs, 30–40% of these patients will eventually progress to liver-related death within a 10-year period [2]. Orthotopic liver transplantation has shown efficacy on the treatment of end-stage liver failure [3] and NASH [4]. However, high cost, donor organ shortage and life-long immunosuppressive medications limit the availability of such treatment [5]. Recently, transplantation of mesenchymal stem cells has shown functional engraftment [6] or remarkable therapeutic efficacy [7] in recipient with induction of NASH, suggesting cell-based therapy as a feasible alternative to ameliorate such manifestations. However, the molecular and cellular mechanisms of NASH are not yet fully understood. Therapeutic strategies that target specific pathways in the pathogenesis of NASH are urgently needed.

Recent progression in induced pluripotent stem cell (iPSC) research has demonstrated that somatic cells can be reprogrammed into a pluripotent state by forced expression of exogenous genes [8–11]. This novel technology has raised the possibility of personalized therapy using patient-specific iPSCs. However, senescence is a critical barrier that may limit reprogramming. To overcome this obstacle, Lapasset et al. has reported a protocol using a combination of six-factor Oct4/Sox2/Klf4/c-Myc/Lin28/Nanog to improve iPSC reprogramming efficiency of senescent fibroblasts from aged donors [12]. In addition, c-Myc, an oncogene causing genomic instability, has been linked to the risk of tumorigenesis, and therefore hinder clinical applications [13]. Although obviation of c-Myc gene reduced the tumorigenic incidence, the reprogramming efficiency was concomitantly suppressed [14, 15]. Expectably, using such c-Myc-free protocol may largely hindered the reprogramming of senescent cells. Collectively, how to efficiently increase reprogramming rate and pluripotency in senile tissues/fibroblasts and to simultaneously obviate the unexpected effect induced by exogenous c-Myc transduction still remained an open question.

Poly(ADP-ribose) polymerase 1 (Parp1) that catalyzes PARylation is a key effector involved in DNA repair, replication, transcription and genomic methylation patterns [16, 17]. It has been indicated that hepatocytes from Parp1-deficient mice have DNA damage and reduced proliferative responses to mitogens [18]. Malfunction of Parp1 signaling may exacerbate diet-induced obesity and insulin insensitivity [19]. These findings suggested Parp1 as a crucial factor in hepatic protection. Recently, Doege et al. and our data have demonstrated that Parp1 can promote reprogramming [20, 21], particularly in the absence of c-Myc gene [21]. Hence, it will be critical to investigate whether Parp1 could also enhance iPSC generation from senescent somatic cells. Whether such resultant iPSCs generated by Parp1-mediated reprogramming can be employed as cell source in the treatment for NASH remain an open question.

In the present study, our findings demonstrated that Parp1 can enhance iPSC generation from murine somatic cells of senescent donors (senescent fibroblasts, SnFBs) in the presence of three other reprogramming factors Oct4/Sox2/Klf4 (OSKP). Parp1-mediated iPSC reprogramming from SnFBs with the OSKP protocol (OSKP-iPSCs) decreased various senescence markers. After induction of hepatic differentiation into hepatocyte-like cells (iPSC-Heps), these OSKP-iPSC-Heps expressed liver-specific markers and characteristics, exhibited mature hepatocyte functions, and were resistance to oxidative insults. Furthermore, intrasplenic transplantation of iPSC-Heps could effectively prevented methionine choline-deficient diet (MCDD)-induced NASH through amelioration of NASH-associated steatosis, oxidative damage and apoptosis. Our data may provide an indication regarding the patient-specific cell therapy for elderly patients with NASH or NASH-associated end-stage liver diseases.

## RESULTS

### Reprogramming SnFBs into OSKP-iPSCs using OSK and Parp1

**Figure 1:**
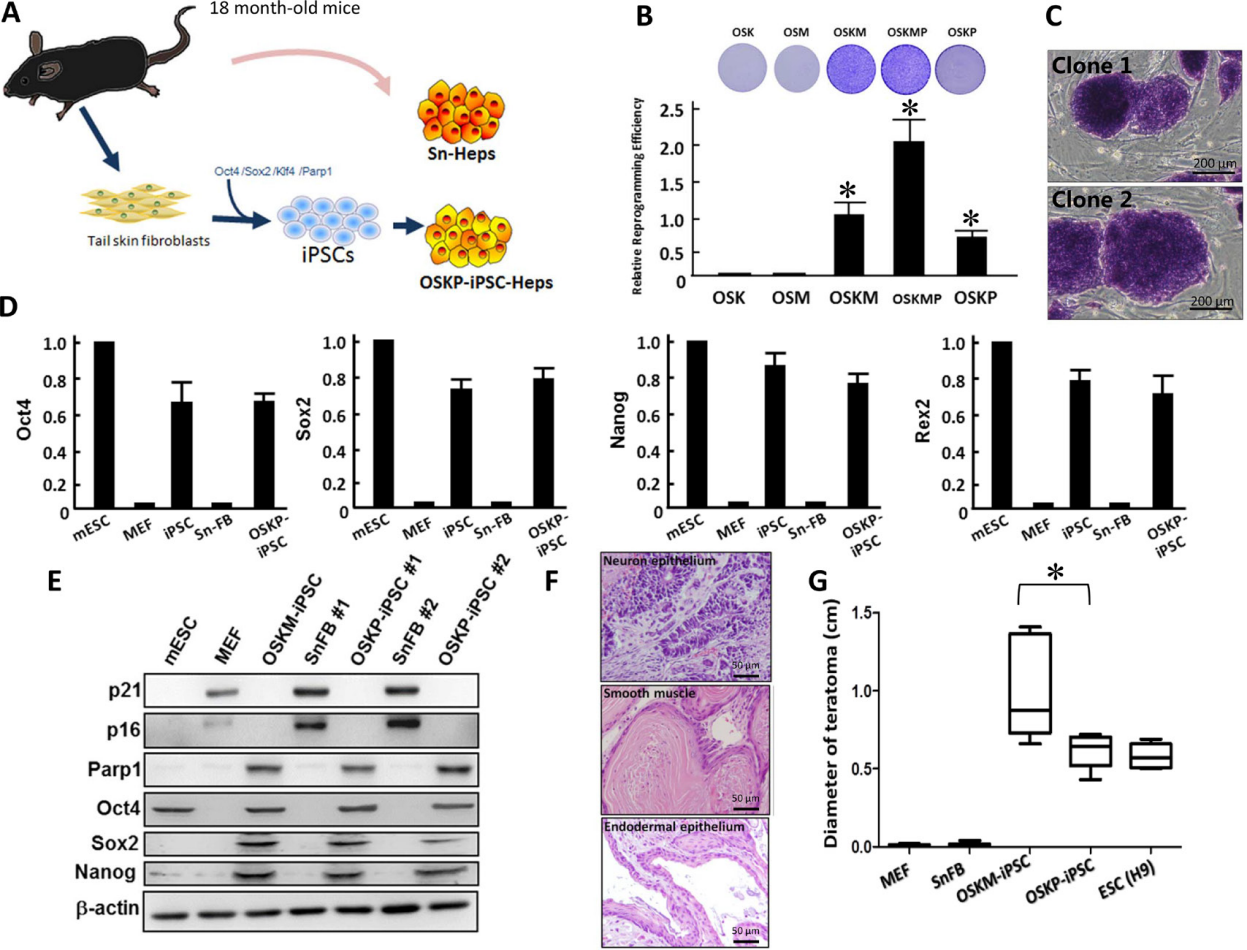
Reprogramming senescent somatic cells into iPSCs using Oct4/Sox2/Klf4 and Parp1 (**A**) Scheme illustrating the isolation of primary hepatocytes and the generation of Oct4/Sox2/Klf4/Parp1-reprogrammed hepatocytes from 18-month-old aging mice. (**B**) Relative reprogramming efficiency among iPSCs generated by different reprogramming factors including Oct4/Sox2/Klf4/Parp1, Oct4/Sox2/Klf4/c-Myc, Oct4/Sox2/Klf4, Oct4/Sox2/c-Myc, and Oct4/Sox2/Klf4/c-Myc/Parp1. (**C**) Representative staining for alkaline phosphatase in Oct4/Sox2/Klf4/Parp1-reprogrammed iPSCs (clones 1 and 2). (**D**) Relative mRNA expression of Oct4, Sox2 and Nanog among mouse embryonic stem cells (mESCs), MEF, Oct4/Sox2/Klf4/c-Myc-reprogrammed iPSCs, senescent fibroblasts, and Oct4/Sox2/Klf4/Parp1-reprogrammed iPSCs. (**E**) Western blotting showing the expression of senescent markers p16 and p21 and stemness markers Oct4, Sox2, and Nanog in two Oct4/Sox2/Klf4/Parp1-reprogrammed iPSC clones. (**F**) Teratoma formation by Oct4/Sox2/Klf4/Parp1-reprogrammed iPSCs. (**G**) Comparison of teratoma size between the recipients of Oct4/Sox2/Klf4/Parp1 and Oct4/Sox2/Klf4/c-Myc. In Panel B, ^*^P < 0.05 vs. OSK or OSM; in panel G, ^*^P < 0.05 vs. OSKM.

Stem cells such as mesenchymal stem cells have been shown to exhibit therapeutic efficacy in experimental models of non-alcoholic steatohepatitis (NASH) [7]. However, it remains an open question whether senescent somatic cells or senescent cell-reprogrammed iPSCs from aged patients could serve as feasible cell source for cell-based therapy in such disease. We have previously demonstrated that Parp1 can promote the reprogramming of somatic cells into iPSCs, particularly in the absence of c-Myc gene [21]. In the present study, we sought to isolate tail skin fibroblasts and reprogrammed them into iPSCs, followed by hepatic differentiation into iPSC-derived hepatocytes (iPSC-Heps). Subsequently, we attempted to compare several hepatic-specific characteristics and therapeutic potential of these iPSC-Heps with senescent hepatocytes isolated from the same cell donor (Figure 1A). To investigate whether Oct4/Sox2/Klf4 plus Parp1 but not c-Myc was able to promote reprogramming of senescent somatic cells, we isolated senescent fibroblasts (SnFBs) from 18-month-old C57B6/L mice, and examined the consequence of SnFBs after treating various reprogramming cocktails that drive iPSC generation. Transfection of Oct4/Sox2/Klf4 (OSK) or Oct4/Sox2/c-Myc (OSM) in SnFBs showed lower reprogramming efficiency than that of conventional cocktail Oct4/Sox2/Klf4/c-Myc (OSKM). OSKM with additional overexpression of Parp1 further enhanced iPSC generation. Notably, overexpression of Parp1 in the presence of three other factors but lacking c-Myc (OSKP) promoted iPSC generation at a moderate magnitude (Figure 1B). These OSKP-reprogrammed iPSCs formed colonies very similar to embryonic stem cells (ESCs) and were positive for alkaline phosphatase (ALP) (Figure 1C). Quantitative RT-PCR showed that, similar to mouse ESCs, OSKP-mediated reprogramming substantially increased the gene expression of stemness factors including Oct4, Sox2, Nanog, and Rex1 (Figure 1D). Western blot further showed that, senescent markers p16 and p21 were abolished in SnFB clones transfected with either OSKP or OSKM, and meanwhile, Parp1, Oct4, Sox2, and Nanog were upregulated in two OSKP-reprogrammed iPSC clones (Figure 1E). Furthermore, OSKP-reprogrammed iPSCs were capable of differentiation into tridermal lineages (mesodermal-lineage, positive for a-smooth muscle actin; neuroectodermal-lineage, positive for nestin; data not shown) and teratoma formation after implantation into subcutaneous space in immune-compromised mice (Figure 1F). Remarkably, the size of teratoma in OSKP recipients was significantly smaller than that in OSKM recipients (Figure 1G). Collectively, these data indicated that Parp1 plus the conventional factors Oct4/Sox2/Klf4 could reprogram senescent somatic cells into pluripotent stem cells.

### Differentiation of OSKP-iPSC-Heps into functional OSKP-Heps

**Figure 2:**
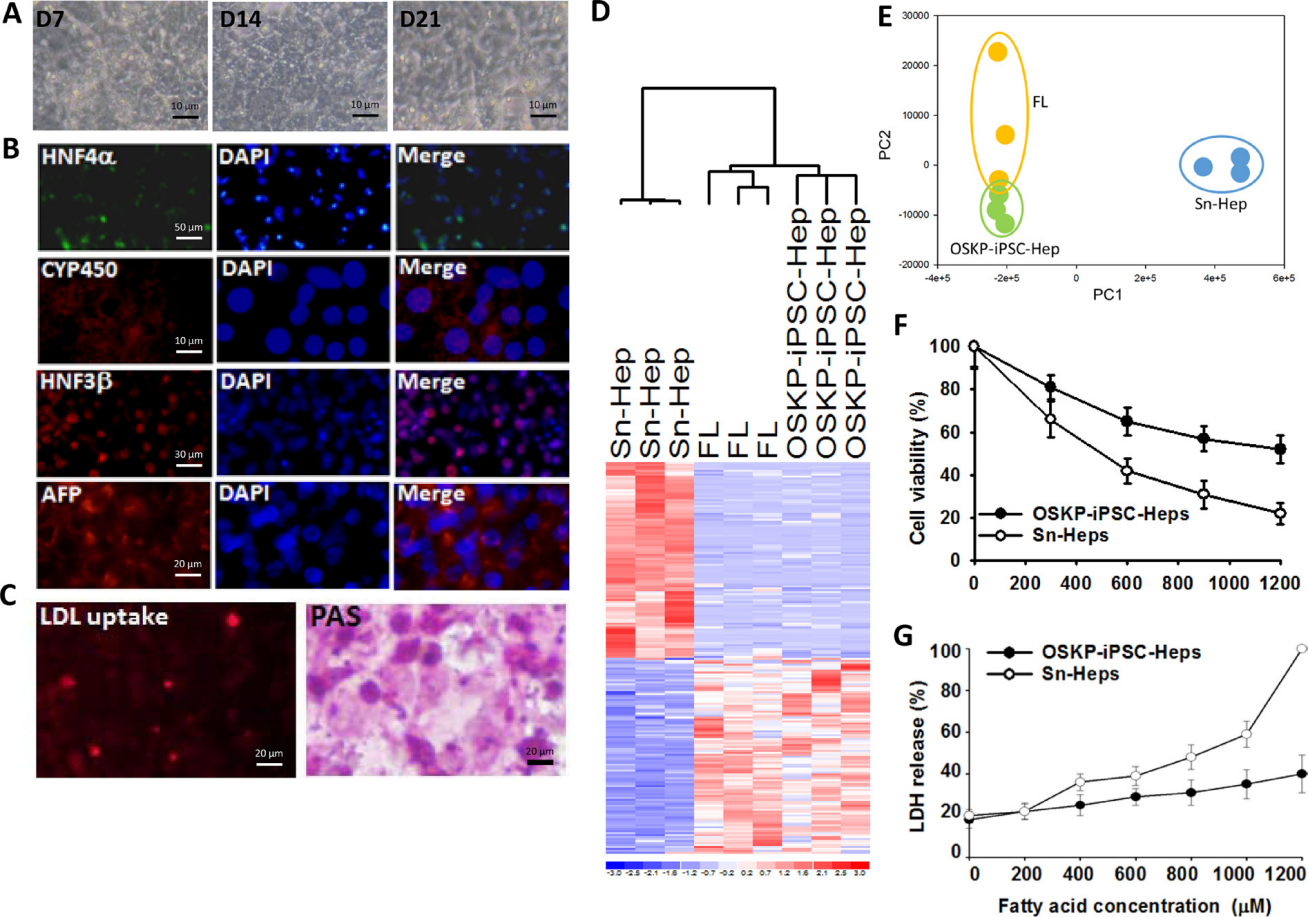
Differentiation of Oct4/Sox2/Klf4/Parp1-reprogrammed iPSCs into hepatocyte-like cells (OSKP-iPSC-Heps) (**A**) Morphology changes of OSKP-iPSC-Heps during the differentiation course. (**B**) Immunofluorescence showing the expression of several hepatic-specific markers, including HNF-3β, alphafetoprotein (AFP), and albumin in OSKP-iPSC-Heps. (**C**) Abilities for LDL uptake and glycogen synthesis in OSKP-iPSC-Heps. (**D**) Microarray analysis revealing the profile of differentially expressed genes among OSKP-iPSC-Heps, senescent primary hepatocytes (Sn-Heps) and fetal liver. (**E**) Multi-dimensional scaling analysis showing the gene expression pattern among OSKP-iPSC-Heps, Sn-Heps and fetal liver. Effect of long-term exogenous fatty acid exposure on (**F**) cell viability and (**G**) LDH release in Sn-Heps and OSKP-iPSC-Heps.

After shifting of OSKP-iPSC-derived embryoid bodies (EBs) to hepatic differentiation media, they gradually exhibited more spread and cuboidal morphology along the differentiation course and eventually differentiated into iPSC-Heps (OSKP-iPSC-Heps; Figure 2A). Immunofluorescence showed that several hepatic-specific markers, including HNF-3β, alphafetoprotein (AFP), and albumin were recruited and reached maximal expression after the 28-day differentiation course (Figure 2B). In addition, these OSKP-iPSC-Heps exhibited regular abilities for LDL uptake and glycogen synthesis (Figure 2C). Microarray analysis further revealed that the profile of differentially expressed genes of OSKP-iPSC-Heps was similar to that of fetal liver, but not senescent liver (Figure 2D). Multi-dimensional scaling analysis further showed that the gene expression pattern of OSKP iPSC-Heps was closer to the patterns of fetal liver than that of Sn-Heps (Figure 2E). To investigate the differential cellular response to exogenous fatty acid treatment and lipid overload in OSKP-iPSC-Heps and Senescent hepatocytes (Sn-Heps), OSKP-iPSC-Heps and Sn-Heps were exposed to various concentrations of fatty acids for 24 hours. Such treatment led to decrease in cell viability and the release of lactate dehydrogenase (LDH) in a dose-related manner in both hepatocytes (Figure 2F and 2G). The maximal dose of exogenous fatty acid (1200mM) resulted in ~40% cell death in OSKP-iPSC-Heps and ~80% cell death in Sn-Heps.

**Figure 3:**
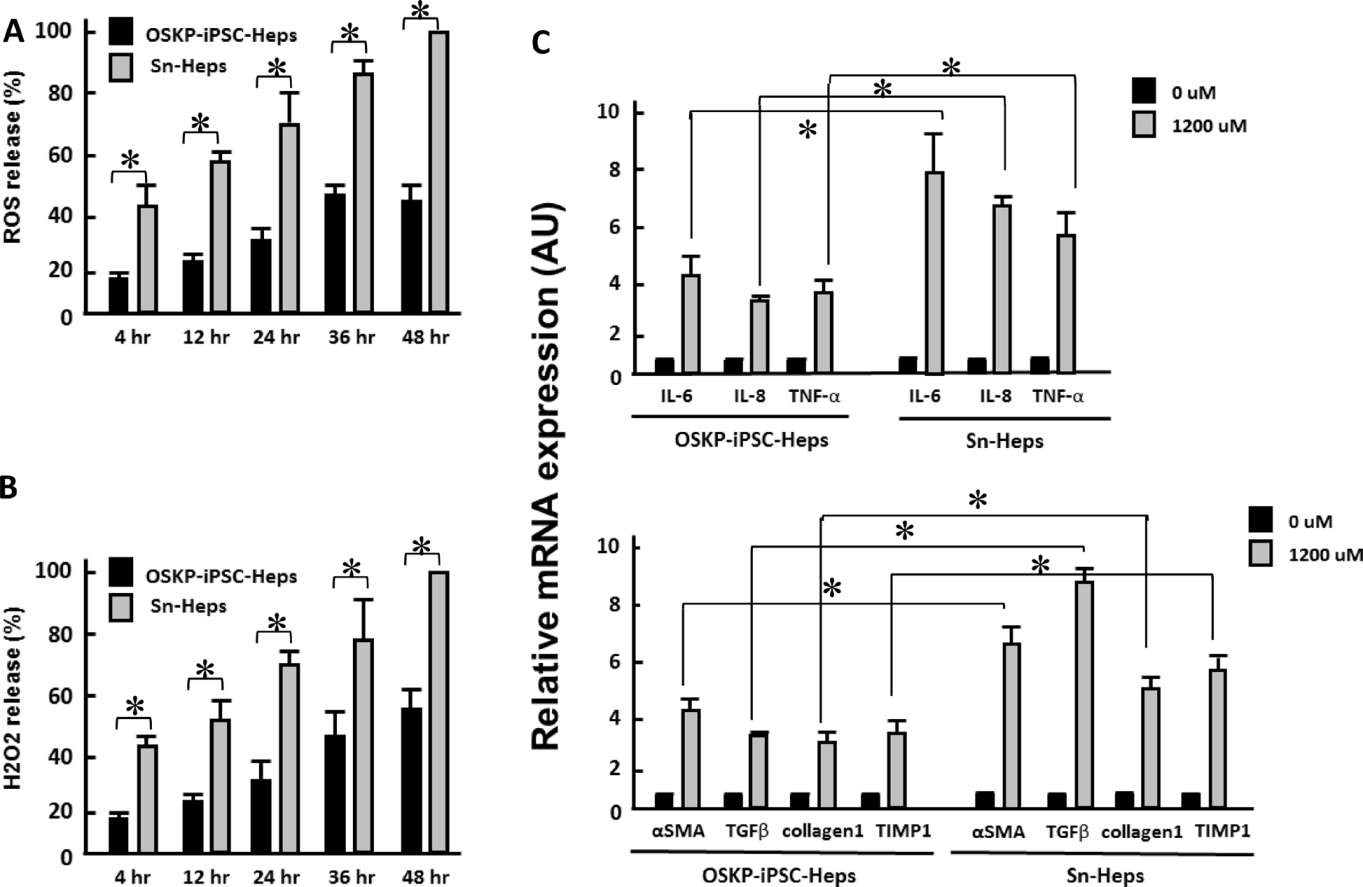
Effect of long-term exogenous fatty acid exposure on the release of oxidative substances and inflammatory and fibrogenic genes Time-dependent effect of long-term exogenous fatty acid exposure on the release of (**A**) ROS and (**B**) H_2_O_2_. Effect of long-term exogenous fatty acid exposure on the expression of (**C**) Upper: inflammatory cytokines IL-6, IL-8 and TNF-α and Lower: fibrogenic genes α-SMA, TGF-β, Collagen1 and Tissue inhibitor of metalloproteinase 1 (TIMP1). In Panels A, B, and C, ^*^P < 0.05 vs. Sn-Heps.

Long-term treatment of exogenous fatty acids led to the production of reactive oxygen species (ROS) and hydrogen peroxide in a time-dependent manner in both hepatocytes, and the production of both oxidative substances were significantly lower in OSKP-iPSC-Heps than that in Sn-Heps (Figure 3A and 3B). Such exogenous fatty acids also elicited a robust mRNA upregulation of several pro-inflammatory cytokines (IL-6, IL-8 and TNF-α; Figure 3C, upper) and fibrosis-associated genes (α-SMA, TGF-β, Collagen1 and Tissue inhibitor of metalloproteinase 1 (TIMP1); Figure 3C, lower) in Sn-Heps, whereas the upregulation of both pro-inflammatory genes and fibrosis-associated genes were restricted in OSKP-iPSC-Heps (Figure 3C). Taken together, these findings demonstrated that OSKP-reprogrammed iPSCs were capable of differentiation into functional hepatocyte-like cells that were less susceptible to exogenous fatty acid challenge than Sn-Heps. The low susceptibility of OSKP-iPSC-Heps to exogenous fatty acids was probably due to the suppressive effect on oxidative substances.

### Resistance of OSKP-iPSC-Heps but not SnFBs to oxidative insults and lipid overload-associated damage

**Figure 4:**
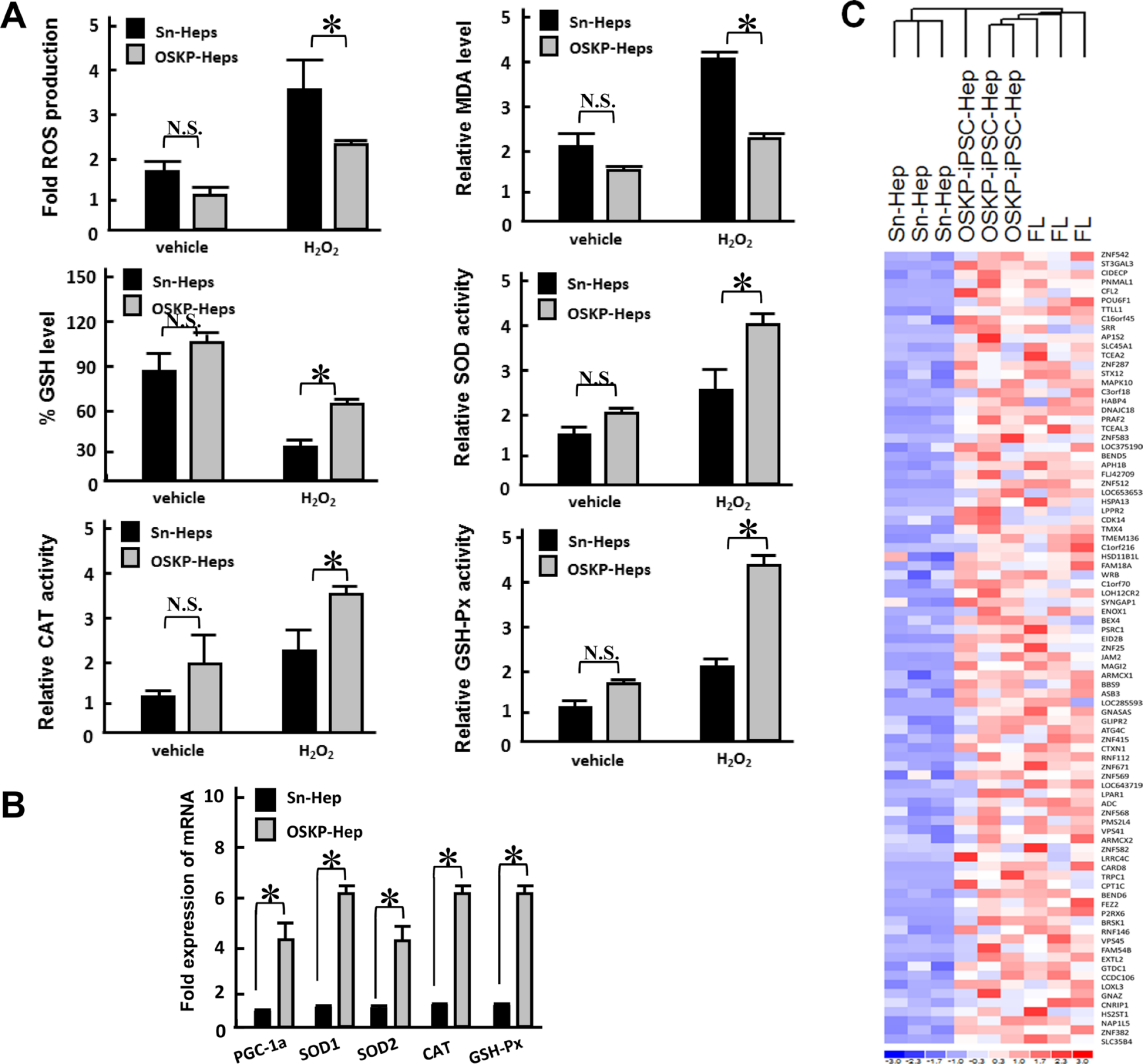
Resistance of OSKP-iPSC-Heps to oxidative insults (**A**) Effect of H_2_O_2_ challenge on the production of ROS (Upper left), malondialdehyde (Upper right), glutathione (Middle left), and the enzyme activity of antioxidant enzymes SOD (Middle right) and CAT (Lower left) and GSH-Px (Lower right) in OSKP-iPSC-Heps and Sn-Heps. (**B**) Quantitative RT-PCR showing the expression of antioxidant genes PGC-1α, SOD1, SOD2, Catalase, and GSH-Px in OSKP-iPSC-Heps and Sn-Heps. (**C**) Gene expression microarray analysis showing the expression profile among OSKP-iPSC-Heps, Sn-Heps and fetal livers. The predominant upregulated processes in OSKP-iPSC-Heps included those related to the antioxidant enzyme system. In Panels A and B, ^*^P < 0.05 vs. Sn-Heps.

Since Parp1 has been linked to DNA repair, longevity and hepatoprotection [18, 22], we next evaluated whether hepatocyte-like cells generated from iPSCs with overexpressing Parp1 but lacking c-Myc could hinder the effect of oxidative insults induced by hydrogen peroxide. There was no significant difference between basal production of ROS and malondialdehyde (MDA, a product of lipid peroxidation), as well as glutathione (GSH), in untreated OSKP-iPSC-Heps and Sn-Heps (Figure 4A). Meanwhile, no difference was also detected between the basal enzyme activity of antioxidant enzymes SOD, CAT, and GSH-Px (Figure 4A). Remarkably, under the challenge of hydrogen peroxide, the production of ROS and MDA were elevated in both Sn-Heps and OSKP-iPSC-Heps, while the production of both substances in OSKP-iPSC-Heps were much lower than that in Sn-Heps (Figure 4A, upper left and right). The levels of GSH were unaffected in stimulated Sn-Heps whereas were inversely increased by hydrogen peroxide in stimulated OSKP-iPSC-Heps (Figure 4A, middle left). The enzyme activities of SOD, CAT and GSH-Px were slightly increased in Sn-Heps by hydrogen peroxide, while were largely elevated in OSKP-iPSC-Heps by the same treatment (Figure 4A, middle right, lower left and right). We further assessed and compared the gene expression of antioxidant enzymes in Sn-Heps and OSKP-iPSC-Heps. Quantitative RT-PCR revealed that the expression of various antioxidant enzymes including PGC-1α, SOD1, SOD2, Catalase, and GSH-Px, consistently tended to be higher in OSKP-iPSC-Heps than those in Sn-Heps (Figure 4B). Furthermore, we used microarray analysis to evaluate and compare the gene expression pattern among fetal liver, Sn-Heps and OSKP-iPSC-Heps. The expression profiles of the differentially expressed genes of OSKP-iPSC-Heps, fetal liver, Sn-Heps based upon their functions in the Gene Ontology database were displayed in Figure 4C. Similar to fetal liver, the predominant upregulated processes in OSKP-iPSC-Heps included those related to antioxidant enzyme system (Figure 4C), suggesting that low levels of oxidative substances in OSKP-iPSC-Heps were attributable to upregulation of several antioxidant enzymes in such hepatocyte-like cells. These findings may provide plausible explanation for the resistance of OSKP-iPSC-Heps to oxidative insults.

### Figure 4 Transplantation of OSKP-iPSC-Heps alleviated steatohepatitis in MCD diet-fed mouse model of NASH

**Figure 5:**
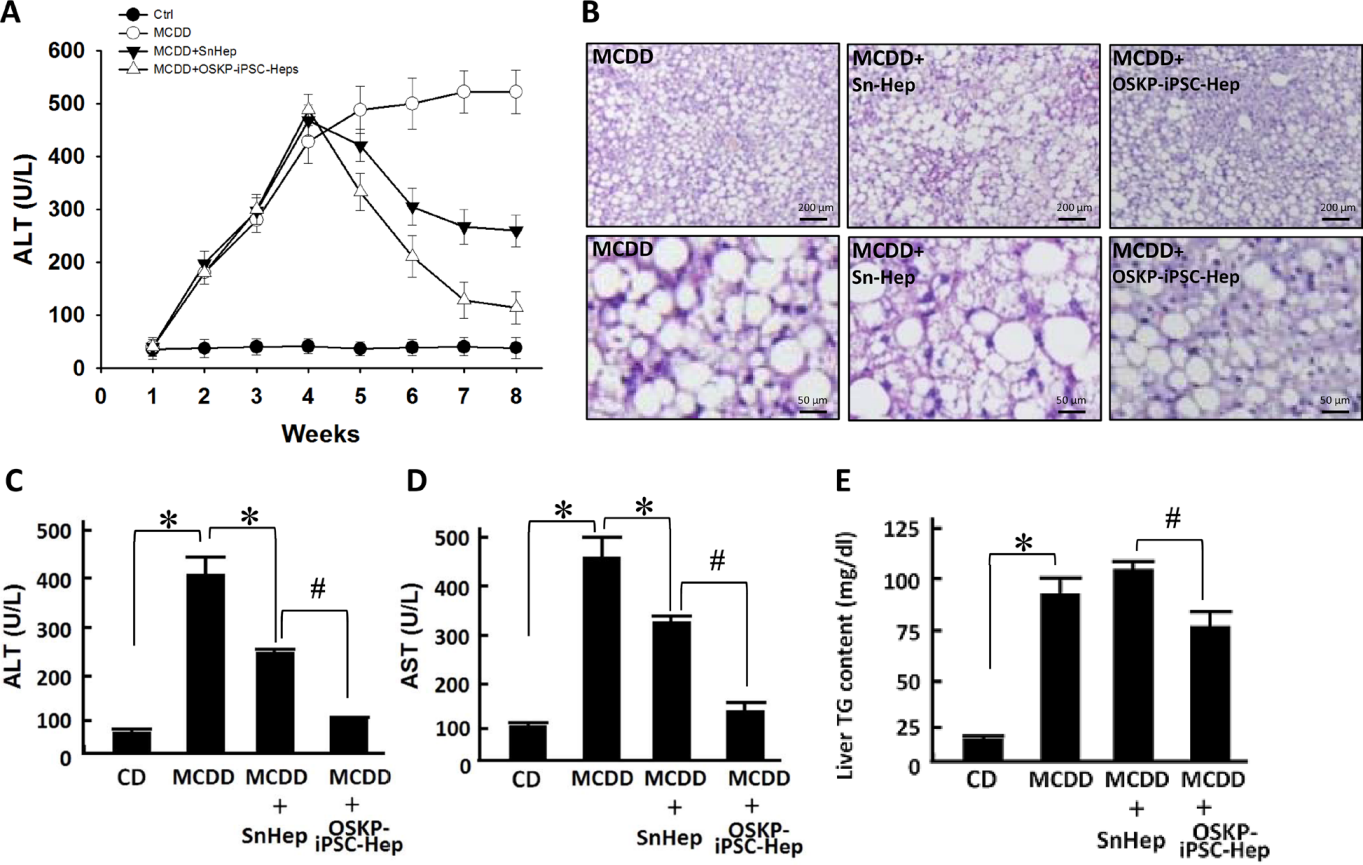
Transplantation of OSKP-iPSC-Heps alleviated steatohepatitis in 18-month-old male MCD diet-fed mouse (**A**) Time course of the changes in serum ALT levels among control diet-fed mice, MCDD-fed mice, and MCDD-fed mice with intrasplenic transplantation of OSKP-iPSC-Heps or SnHeps. (**B**) H & E staining showing the fat accumulation and severe hepatocyte vacuolization over the liver from MCDD-fed mice. Effect of intrasplenic transplantation of OSKP-iPSC-Heps or SnHeps on serum (**C**) ALT and (**D**) AST levels, and (**E**) hepatic triglyceride accumulation in MCDD-fed mice. In Panels C, D and E, ^*^P < 0.05 vs. MCDD; ^#^*P* < 0.05 vs. MCDD+SnHep.

**Figure 6:**
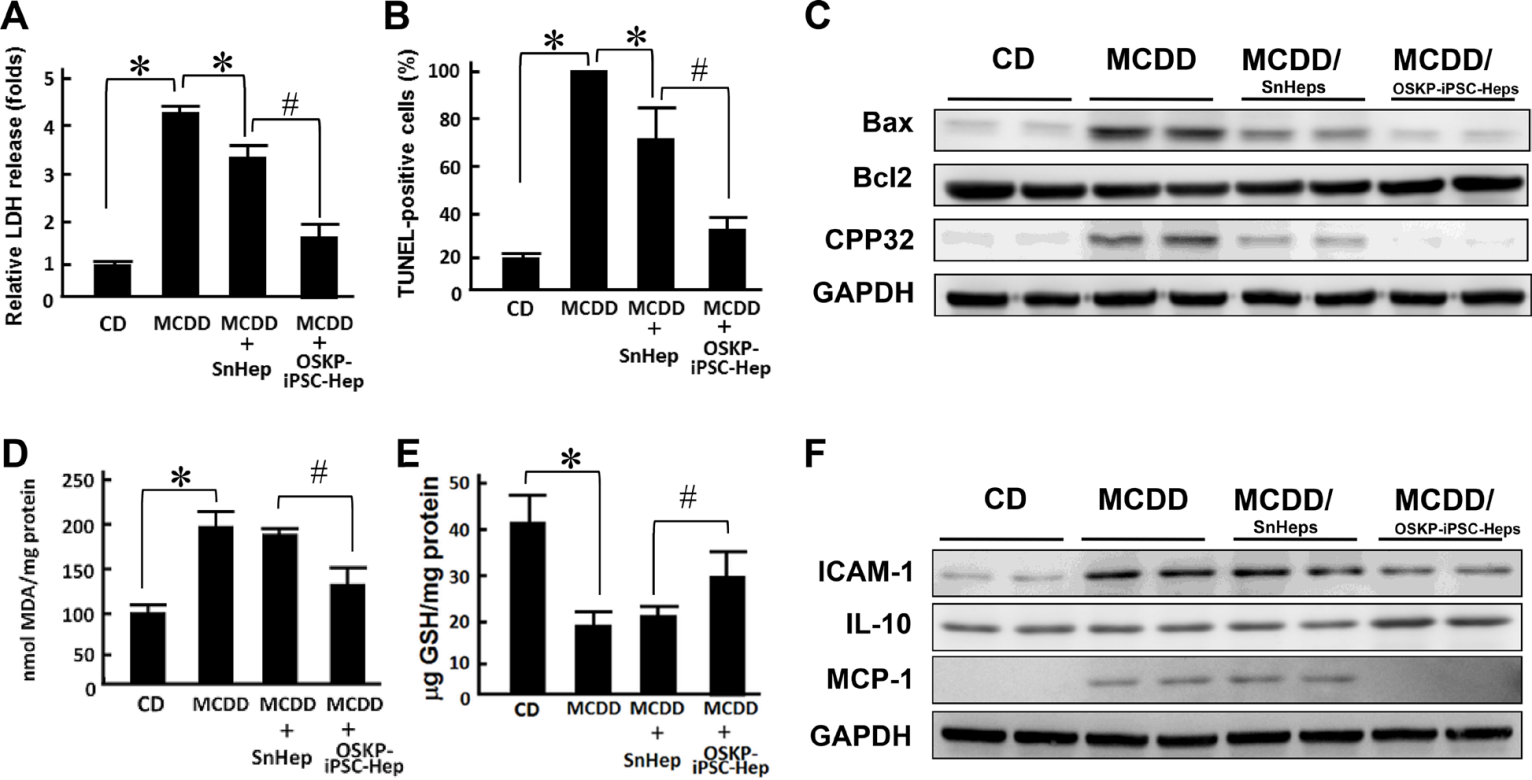
Transplantation of OSKP-iPSC-Heps alleviated the production of oxidative substances and cell apoptosis Effect of intrasplenic transplantation of OSKP-iPSC-Heps or SnHeps on (**A**) the release of LDH and (**B**) the numbers of hepatic apoptotic cells. (**C**) Western blotting showing the protein expression of Bax, Bcl2, and CPP32 in MCDD-fed mice with intrasplenic transplantation of OSKP-iPSC-Heps or SnHeps. Effect of intrasplenic transplantation of OSKP-iPSC-Heps or SnHeps on the hepatic content of (**D**) malondialdehyde and (**E**) GSH. (**F**) Western blotting showing the protein expression of ICAM, IL-10, and MCP-1 in MCDD-fed mice with intrasplenic transplantation of OSKP-iPSC-Heps or SnHeps. In Panels A, B, D and E, ^*^P < 0.05 vs. MCDD; ^#^*P* < 0.05 vs. MCDD+SnHep.

Previous reports have provided evidence demonstrating the functional engraftment [6] and therapeutic potential [7] of stem cells in experimental model of NASH. Our *in vitro* findings indicated that OSKP-iPSC-Heps were more resistant to the challenge of exogenous fatty acids and oxidative insults due to the upregulated anti-oxidant system. To evaluate the therapeutic utility of OSKP-iPSC-Heps *in vivo*, we induced a mouse model of NASH by feeding 18-month-old male C57BL/6J mice with methionine-choline-deficient diet (MCDD) for six weeks and assessed the efficacy of transplanted OSKP-iPSC-Heps on several parameters in the liver of such model. Mouse OSKP-iPSC-Heps or SnHeps were transplanted via an intrasplenic route at 4 weeks post-MCDD feeding, and all parameters were recorded at the end of 6 weeks post-MCDD feeding. Induction NASH by MCDD feeding was evaluated by H&E staining and biochemistry analysis. In MCDD-fed mice, serum levels of ALT were substantially elevated at 4 weeks post-MCDD feeding and persisted for more than 4 weeks. SnHeps only moderately decreased the serum ALT levels while OSKP-iPSC-Heps largely suppressed the levels (Figure 5A). A maximal suppression of MCDD-elicited ALT levels by OSKP-iPSC-Heps was observed at 4 weeks post-transplantation (8-weeks post-MCDD feeding; Figure 5A). Apparent fat accumulation and severe hepatocyte vacuolization over the liver (Figure 5B) and elevated serum levels of the enzymes aspartate transaminase and alanine transaminase (AST and ALT, Figure 5C and 5D), triglyceride overaccumulation (Figure 5E) were observed in MCDD-fed mice. Intrasplenic transplantation of SnHeps for two weeks showed no obvious effect on the diffuse hepatic steatosis while mildly attenuated the serum ALT and AST levels in MCDD-fed mice (Figure 5A–5D). Remarkably, transplantation of OSKP-iPSC-Heps attenuated the steatosis and turned the macrovacuolar type of steatosis into a predominantly microvacuolar type in MCDD-fed mice (Figure 5B). The accumulation of liver triglycerides was unaffected by Sn-Heps but was moderately reduced by OSKP-iPSC-Heps (Figure 5E). Elevation of serum LDH levels and TUNEL-positive cells in the steatotic liver were detected in MCDD-fed mice (Figure 6A and 6B), but not in the mice fed with control diet. Notably, LDH release and the mean TUNEL-positive cells were not modified by Sn-Heps but were largely suppressed by the transplantation of OSKP-iPSC-Heps (Figure 6A and 6B). Along with the observations in TUNEL assay, we further evaluated the Bax/Bcl2 ratio and caspase 3 (CPP32) expression in the steatotic liver tissues with or without indicated transplantation. A prominent elevation of the Bax/Bcl-2 ratio and CPP32 expression was detected in livers from the MCDD-fed mice (Figure 6C). This elevation of Bax/Bcl-2 ratio and CPP32 expression was slightly reduced by Sn-Heps and was substantially decreased by OSKP-iPSC-Heps (Figure 6C).

One of the main hypothetical mechanisms underlying NASH-related hepatocyte injury is the fatty acid-elicited oxidative stress and lipid peroxidation [23]. Considering the higher antioxidant activity of OSKP-iPSC-Heps than that of SnHeps, we compared the MDA and GSH levels in the liver samples from recipients of OSKP-iPSC-Heps and SnHeps. In MCDD-fed recipients, basal hepatic lipoperoxide levels were significantly increased. This elevation of MDA levels was not affected by transplantation by SnHeps, but was largely suppressed by OSKP-iPSC-Heps (Figure 6D). Reduction of glutathione (GSH), a well-known endogenous antioxidant, was detected in liver samples from MCDD-fed mice. Transplantation of OSKP-iPSC-Heps but not SnHeps moderately restored GSH levels in the liver sample in MCDD-fed mice (Figure 6E). Furthermore, the expression of adhesion molecule ICAM-1, pro-inflammatory cytokine MCP-1 and anti-inflammatory cytokine IL-10 in the steatotic liver were measured. As detected by Western blot, a significant increase in ICAM-1 and MCP-1 protein content was observed in the livers from MCDD-fed mice, which was suppressed by the transplantation of OSKP-iPSC-Heps (Figure 6F). The anti-inflammatory cytokine IL-10 remained not changed after Sn-Hep transplantation but was inversely increased by OSKP-iPSC-Hep transplantation (Figure 6F). Collectively, these findings demonstrated the transplantation efficacy of OSKP-iPSC-Heps that ameliorates steatosis, liver functions, hepatocyte apoptosis, pro-inflammatory changes and the accumulation of oxidative stress in MCDD-fed mouse model of NASH.

## DISCUSSION

The pathogenesis of NASH has not yet been fully elucidated. Many patients with steatosis never develop necro-inflammation or fibrosis [24], indicating that the development of steatohepatitis requires some other factors apart from steatosis. The “two-hit hypothesis” proposed by Day in1998 have depicted that the first ‘hit’ steatosis increases the sensitivity of the liver to the second ‘hit’ that mediates liver injury, including oxidative stress, cytokines, etc [23]. Direct regulatory effect of exogenous fatty acid treatment on mitochondrial electron flux [25], ROS generation [26, 27], pro-inflammatory and fibrogenic cytokines [27, 28] in cultured hepatoma cells or primary hepatocytes have been reported previously. In this study, our data showed that mouse Oct4/Sox2/Klf4/Parp1-reprogrammed iPSC-Heps (OSKP-iPSC-Heps) exhibited higher antioxidant defensive activity than senescent primary hepatocytes. Consistent with previous reports [25–28], chronic exposure of both OSKP-iPSC-Heps and SnHeps to fatty acid also increased cytotoxicity, oxidative stress, proinflammatory and firbogenic cytokines. However, mouse OSKP-iPSC-Heps appeared to be more resistant to these insults. Comparing with that of senescent primary hepatocytes, intrasplenic transplantation of mouse OSKP-iPSC-Heps exhibited a better efficacy that effectively ameliorated the steatosis and simultaneously improved hepatic functions and reduced oxidative stress, pro-inflammatory cytokines and fibrogenic changes. By contrary, senescent primary hepatocytes only carried a mild efficacy on biochemical parameters of liver functions, LDH release and apoptosis, and showed no detectable effect on the accumulation of triglyceride and MDA, and the restoration of glutathione in the livers from MCDD-fed mice.

Senescence is characterized by an irreversible cell cycle arrest under various stresses, e.g. oncogene activation, shortened telomeres [29]. Cellular reprogramming resets the epigenetic landmarks and has the ability to counteract the mechanisms of cellular senescence and bring the cells to a self-renewing and rejuvenated state. By forced expression of reprogramming factors, somatic cells could be reprogrammed into induced pluripotent stem cells (iPSCs), which possess potential for self- renewal and multi-lineage differentiation [8–11] and have been regarded as alternative resources for restorative cell therapy. An elegant report by Lapasset et al. have demonstrated that manipulation of induction protocol Oct4/Sox2/Klf4/c-Myc with transcription factors Nanog and Lin28 could reprogram centenarian somatic cells into iPSCs [12]. c-Myc, an oncogene that causes genomic instability, has been linked to the risk of tumorigenesis, and therefore hinder the clinical applications of conventional iPSCs [13]. Although obviation of exogenous c-Myc gene did substantially decrease tumorigenic incidence, the reprogramming efficiency was simultaneously suppressed [14, 15]. Therefore, these findings and evidence revealed that a modified protocol will be required for iPSC generation from senescent somatic cells.

Parp1 catalyzes PARylation and is a key effector involved in DNA repair, replication, transcription and genomic methylation patterns [16, 17, 30]. In addition, Parp1 has been shown to regulate telomere length [31] and modulate telomerase activity by altering PARylation of telomerase reverse transcriptase (TERT) [32]. Recently, it was reported that Parp1 contributes to early-stage epigenetic modification during somatic cell reprogramming [20], and can promote reprogramming and maintain pluripotency [21]. In this study, our findings have shown that a four-factor cocktail (Oct4/Sox2/Klf4/Parp1; OSKP) could successfully reprogram senescent cells from aged mice into iPSCs in the absence of exogenous c-Myc gene. The resultant OSKP-iPSCs could undergo hepatic-specific differentiation and generate OSKP-iPSC-Heps, which exhibited a better hepatoprotective efficacy than senescent primary hepatocytes to improved MCD diet-induced steatohepatitis. The mechanisms for OSKP-iPSC-Heps that ameliorated MCD diet-induced steatohepatitis were not clear. One possible mechanism could be that the transplanted OSKP-iPSC-Heps with mature hepatocyte characteristics may compensate the impaired hepatocyte function in the steatotic livers from MCD diet-fed mice. Another possible mechanism could be that OSKP-iPSC-Heps ameliorated the steatohepatitis predominantly by reduction of oxidative stress in this model. It has been proposed that oxidative stress may serve a central role in the pathogenesis of Non-alcoholic fatty liver disease [33–35]. The fat overload in the mitochondria may lead to reduced b-oxidation and ROS over-accumulation, and further induce the lipid peroxidation [36, 37]. Our findings supported the pivotal role of oxidative stress in the pathogenesis of NASH and that intervention of oxidative stress production may improve NASH.

**Figure 7:**
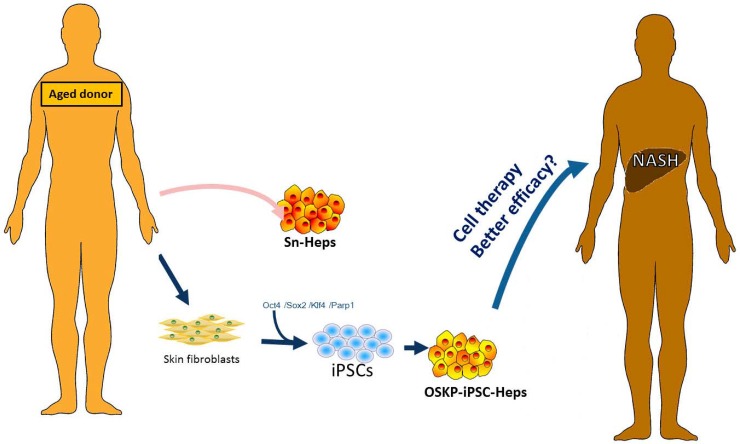
Scheme for the generation of OSKP-iPSC-Heps from aged donor-derived somatic cells Scheme illustrates the feasibility of generation of OSKP-iPSC-Heps from aged donor-derived somatic cells. Such iPSC-based cell therapy may provide better efficacy than senescent primary hepatocytes.

In the present study, our finding indicated that, transfection with Oct4/Sox2/Klf4/Parp1(OSKP) were capable of the generation of OSKP-iPSCs that formed smaller teratoma than iPSCs reprogrammed with the conventional reprogramming factors Oct4/Sox2/Klf4/c-Myc. After hepatic-specific differentiation, OSKP-iPSC-Heps expressed a prominent anti-oxidant system that could effectively scavenge the accumulation of oxidative substances and further ameliorate the steatohepatitis. The treatment efficacy of OSKP-iPSC-Heps was much higher than senescent primary hepatocytes isolated from aged mice. These findings indicated that, comparing with senescent primary hepatocytes from aged donors, an Oct4/Sox2/Klf4/Parp1-reprogrammed iPSCs from senescent cells may be a better cell source for the treatment of NASH (Figure 7). However, more data related to reprogramming senescent human cells and the patient response to transplanted iPSC-differentiated cells will be required to elucidate the clinical utilities of patient-specific iPSC-based therapy in aged patient cohorts.

## MATERIALS AND METHODS

### Generation of iPSC cell lines and differentiation protocols

Murine iPSCs were generated from mouse tail skin fibroblasts derived from 18-month-old C57/B6 mice. The iPSCs were reprogrammed by the transduction of retroviral vectors encoding four transcription factors (Oct-4/Sox2/Klf4/c-Myc; OSKM), three transcription factors (Oct-4/Sox2/Klf4; OSK), or three factors plus parp1 (Oct-4/Sox2/Klf4/Parp1, abbreviated as OSK-P), as described previously [15]. Total of 12 clones (Re-1 to Re-12; OSKM) were selected and established. The clone of Re-7 iPSCs had been stably passed to more than 100th passages with high pluripotency. Therefore, Re-7 iPSCs were selected and widely used in this study. Briefly, undifferentiated iPSCs were routinely cultured and expanded on mitotically-inactivated MEFs (50,000 cells/cm^2^) in six-well culture plates (BD Technology) in the presence of 0.3% leukemia inhibitory factor in an iPSC medium consisting of Dulbecco's Modified Eagle's Medium (DMEM; Sigma) supplemented with 15% fetal bovine serum (FBS; Invitrogen), 100 mM minimal essential medium (MEM) nonessential amino acids (Sigma), 0.55 mM 2-mercaptoethanol (Gibco), and antibiotics (Invitrogen), and 0.3% leukemia inhibitory factor (Abcam). Every three to four days, colonies were detached with 0.2% collagenase IV (Invitrogen), dissociated into single cells with 0.025% trypsin (Sigma-Aldrich) and 0.1% chicken serum (Invitrogen) in PBS, and replated onto MEFs. For embryoid body (EB) formation, iPSCs were dissociated into a single cell suspension by 0.25% trypsin-EDTA and plated onto non-adherent culture dishes in DMEM with 15% FBS, 100 mM MEM nonessential amino acids, 0.55 mM 2-mercaptoethanol, and antibiotics at a density of 2 × 10^6^ cells/100 mm plate. After 4 days in floating culture, EBs were transferred onto gelatin-coated plates and maintained in the same medium for 24 h. EBs were then assigned for *in vitro* hepatocyte differentiation by using a two-step procedure as previously described [15]. For endoderm induction, iPSCs were incubated for 24 h in RPMI 1640 medium (Invitrogen/Gibco, Rockville, MD, USA), supplemented with 100 ng/ml Activin A (Peprotech). On the following 2 days, 0.1 and then 1% insulin-transferrin-selenium (Invitrogen/Gibco) was added to this medium. Following Activin A treatment, the differentiated iPSCs were cultured in Hepatocyte Culture Medium (HCM) (Cambrex, Baltimore, MD, USA) containing 30 ng/ml FGF4 for 4 days. Then, the differentiated cells were incubated in HCM containing 20 ng/ml HGF for 6 days, in HCM containing 10 ng/ml oncostatin-M (R&D, Minneapolis, MN, USA) plus 0.1 mM dexamethasone (SigmaeAldrich) for 5 days.

### Induction of non-alcoholic steatohepatitis and isolation of primary hepatocytes

Non-alcoholic steatohepatitis was induced by continuous feeding of methionine-choline-deficient diet (MCDD) for six weeks in 18-month-old male C57BL/6J mice, as reported previously with brief modifications [6]. The therapeutic effect of senescent primary hepatocytes (SnHeps) and iPSC-derived hepatocytes (iPSC-Heps) generated from the 18-month-old aging donors on mice with non-alcoholic steatohepatitis were compared. We generated murine iPSCs from mouse tail skin fibroblasts, and then differentiate these iPSCs into hepatocyte-like cells (iPSC-Heps; please refer to the methods Generation of iPSC cell lines and differentiation protocols). The primary cultured hepatocytes were isolated from livers of senescent mice through a collagenase perfusion method, and plated on six-well culture plates, as previously described [38, 39]. After 4 weeks post-MCDD feeding, MCDD-fed mice were transplanted with senescent primary hepatocytes (Sn-Heps) or OSKP-iPSC-Heps via an intra-splenic route. All parameters were recorded at 6 and 8 weeks post-MCDD feeding. Induction of non-alcoholic steatohepatitis was confirmed by H&E staining and biochemistry analysis. Meanwhile, parameters from recipients of Sn-Heps or OSKP-iPSC-Heps were compared to evaluate the efficacy of cell transplantation.

### Real-Time reverse transcription-polymerase chain reaction (RT-PCR)

Real-time RT-PCR was performed as previously described [40]. For real-time RT-PCR analysis, the total RNA of cells was extracted using the RNAeasy kit (Qiagen, Valencia, CA). Briefly, the total RNA (1 μg) from each sample was reversely transcribed using 0.5 μg of oligo dT and 200 U Superscript II RT (Invitrogen, Carlsbad, CA). The amplification was carried out in a total volume of 20 μl containing 0.5 μM of each primer, 4 mM MgCl2, 2 ml LightCycler FastStart DNA Master SYBR green I (Roche Diagnostics, Pleasanton, CA) and 2 ml of 1:10 diluted cDNA. The quantification of the unknown samples was performed by LightCycler Relative Quantification Software, version 3.3 (Roche Diagnostics). In each experiment, the GAPDH housekeeping gene was amplified as a reference standard. PCR reactions were prepared and performed in duplicate and heated to 95°C for 10 minutes followed by 40 cycles of denaturation at 95°C for 10 seconds, annealing at 55°C for 5 seconds, and 40 cycles of extension at 72°C for 20 seconds. Standard curves (cycle threshold values versus template concentration) were prepared for each target gene and for the endogenous reference (GAPDH) in each sample.

### Teratoma formation and histological analysis

5 × 10^6^ iPSCs in 100 μL of PBS were subcutaneously injected into NOD/SCID mice via a 21 G needle. Teratomas were surgically removed after 3–4 weeks and fixed in formalin at 4°C overnight, embedded in paraffin wax, and sectioned. Sections were stained with haematoxylin and eosin for pathological examination.

### Histological survey for teratoma

iPSCs were suspended at 1 × 10^7^ cells/ml in DMEM containing 10% FBS. Nude mice were anesthetized with diethyl ether. We injected 100 ml of the cell suspension (1 × 10^6^ cells) subcutaneously into the dorsal flank. Four weeks after the injection, tumors were surgically dissected from the mice. Samples were weighed, fixed in PBS containing 4% formaldehyde, and embedded in paraffin. Sections were stained with hematoxylin and eosin.

### Cellular uptake assay of low-density lipoprotein (LDL)

The uptake capability of 1,1’-dioctadecyl-1-,3,3,3’,3’,-tetramethyl-indo-carbocyanine perchlorate conjugated to acetylated-LDL (DiI-Ac-LDL; AbD Serotec) of cells was determined by fluorescent microscopy. Cells were incubated with 20 μg/ml DiIAC-LDL at 37°C for 24 h. Incorporation of DiI-Ac-LDL into cells was visualized by fluorescence microscopy.

### Periodic Acid-Schiff (PAS) staining for glycogen

Cells in culture dishes were fixed in 4% paraformaldehyde, permeabilized with 0.1% Triton X-100 for 10 min. Cells in culture dishes were then oxidized in periodic acid (Sigma-Aldrich) for 5 min at room temperature, rinsed three times in distilled water, treated with Schiff's reagent (Sigma-Aldrich) for 15 min at room temperature, and washed in running tap water for 5 minutes. Samples were counterstained with Hematoxylin Solution for 90 seconds.

### MTT assay

For evaluation of cell survival, cells were seeded on 24-well plates at a density of 2 × 10^4^ cells/well, followed by the addition of methyl thiazol tetrazolium (MTT; Sigma) at the end of cell culture. The amount of MTT formazan product was determined using a microplate reader at an absorbance of 560 nm (SpectraMax 250, Molecular Devices, Sunnyvale, CA, USA).

### Measurement of apoptotic cells

Apoptotic cells in the liver were identified by terminal deoxynucleotidyl transferase (TdT)-mediated digoxigenindeoxyuridine nick-end labeling (TUNEL) staining. The TUNEL method for the *in situ* apoptotic assay was performed according to the method of Lee et al. with minor modifications [41]. TUNEL-negative controls were stained without TdT enzyme.

### Immunofluorescence staining

An avidin-biotin complex-based method was used for immunohistochemical staining of differentiated iPSCs. Following washes with 3% hydrogen peroxide, sodium azide and antigenicities were retrieved using a microwave. Each slide was then treated with antibodies for HNF-3ß (Chemicon International, Temecula, CA), Albumin (Chemicon International, Temecula, CA), AFP (Upstate Biotechnology, Waltham, MA, USA). Immunoreactive signals were detected with a mixture of biotinylated rabbit anti-mouse IgG and Fluoresave (Calbiochem, La Jolla, CA, USA) and a confocal microscope (Olympus, FV300).

### Microarray analysis and bioinformatics

Total RNA was extracted from cells using Trizol reagent (Life Technologies, Bethesda, MD, USA) and the Qiagen RNAeasy (Qiagen, Valencia, CA, USA) column for purification. Total RNA was reverse-transcribed with Superscript II RNase H-reverse transcriptase (Gibco BRL) to generate Cy3- and Cy5-labeled (Amersham Biosciences Co., Piscataway, NJ, USA) cDNA probes for the control and treated samples, respectively. The labeled probes were hybridized to a cDNA microarray containing 10,000 gene clone immobilized cDNA fragments. Fluorescence intensities of Cy3 and Cy5 targets were measured and scanned separately using a GenePix 4000B Array Scanner (Axon Instruments, Burlingame, CA, USA). Data analysis was performed using GenePix Pro 3.0.5.56 (Axon Instruments, USA) and GeneSpring GX 7.3.1 software (Agilent, Palo Alto, CA). The average-linkage distance was used to assess the similarity between two groups of gene expression profiles as described below. The difference in distance between two groups of sample expression profiles to a third was assessed by comparing the corresponding average-linkage distances (the mean of all pair-wise distances (linkages) between members of the two groups concerned). The error of such a comparison was estimated by combining the standard errors (the standard deviation of pair-wise linkages divided by the square root of the number of linkages) of the average-linkage distances involved. Classical multidimensional scaling (MDS) was performed using the standard function of the R program to provide a visual impression of how the various sample groups are related.

### Liver functional tests

Biochemical parameters were measured using standard clinical methods. After anesthesia by ketamine (10 mg/100g body wt), intracardiac aspiration of blood was performed. 0.8–0.9 ml of blood sample was collected from the heart into a pyrogen-free syringe containing ~75 units of heparin sodium. Serum ammonia and biochemical parameters, including alanine aminotransferase (ALT), aspartate aminotransferase (AST), and total bilirubin (TBIL) were analyzed using a Vitro DT chemistry system (Johnson & Johnson).

### Statistical analysis

The results are expressed as mean ± SD. Statistical analyses were performed using the *t*-test for comparing two groups, and one-way or two-way ANOVA, followed by Bonferroni's test, to detect differences among three or more groups. The results were considered statistically significant at *P* < 0.05.
